# Effects of Different Vegetation Zones on CH_**4**_ and N_**2**_O Emissions in Coastal Wetlands: A Model Case Study

**DOI:** 10.1155/2014/412183

**Published:** 2014-04-29

**Authors:** Yuhong Liu, Lixin Wang, Shumei Bao, Huamin Liu, Junbao Yu, Yu Wang, Hongbo Shao, Yan Ouyang, Shuqing An

**Affiliations:** ^1^College of Environment and Resources, Inner Mongolia University, Hohhot 010021, China; ^2^Key Laboratory of Coastal Zone Environmental Processes, Yantai Institute of Coastal Zone Research, Chinese Academy of Sciences, Yantai 264003, China; ^3^College of Life Sciences, Inner Mongolia University, Hohhot 010021, China; ^4^The Institute of Wetland Ecology, School of Life Science, Nanjing University, Nanjing 210093, China

## Abstract

The coastal wetland ecosystems are important in the global carbon and nitrogen cycle and global climate change. For higher fragility of coastal wetlands induced by human activities, the roles of coastal wetland ecosystems in CH_4_ and N_2_O emissions are becoming more important. This study used a DNDC model to simulate current and future CH_4_ and N_2_O emissions of coastal wetlands in four sites along the latitude in China. The simulation results showed that different vegetation zones, including bare beach, *Spartina* beach, and *Phragmites* beach, produced different emissions of CH_4_ and N_2_O in the same latitude region. Correlation analysis indicated that vegetation types, water level, temperature, and soil organic carbon content are the main factors affecting emissions of CH_4_ and N_2_O in coastal wetlands.

## 1. Introduction

Methane (CH_4_) and nitrous oxide (N_2_O) are important active greenhouse gases contributing to global warming [1, 2]. Contributions of CH_4_ and N_2_O to global warming are 21 and 310 times of CO_2_ [3] and occupy about 15% and 5% of greenhouse effects [4], respectively. Moreover, the two kinds of gases in the atmosphere are growing at 3% and 0.22% per year, respectively [5]. As an important source and sink of greenhouse gases, the coastal wetland ecosystems are important in the global carbon and nitrogen cycle and global climate change. Since coastal wetlands belong to the ecologically fragile zone [6], it is important to understand the relationships between vegetation characteristics and CH_4_ and N_2_O emissions.

Many studies on CH_4_ and N_2_O emissions in natural wetlands are carried out since the 1990s and focus on their emissions, absorptions, spatial and temporal variations, and environmental factors. Although the effects of vegetation features on CH_4_ and N_2_O emissions from wetland ecosystems worldwide have been investigated (e.g., [7–9]), these studies in our country are still relatively weak. In China, greenhouse gas emission flux and the effects of environmental factors are mainly concentrated on* Phragmites *wetland of the Sanjiang plain [10] and in the southern mangrove coastal wetlands [11, 12]. Recently, the spatial and temporal variations of N_2_O and CH_4_ fluxes associated with abiotic sediment parameters are quantified in the coastal marsh dominated by* Suaeda salsa* in the Yellow River estuary [13], and the effects of* different vegetation, Spartina alterniflora* and* Phragmites australis,* on CH_4_ and N_2_O emissions are investigated by using experimental mesocosms [9]. However, these studies do not compare roles of vegetation zone in different areas in CH_4_ and N_2_O emissions and future variations of CH_4_ and N_2_O emissions in coastal wetlands of China.

In this study, denitrification-decomposition (DNDC) model was used to simulate wetland biogeochemistry processes and its response to global warming in the four sites of coastal zone distributing along the latitude. By simulation analysis, the following research questions were focused on the following.

1. Are there differences in effects of different vegetation zones on CH_4_ and N_2_O emissions of coastal wetlands in different sites along latitude?
2. How will CH_4_ and N_2_O emissions change with increasing temperature in coastal wetlands?

## 2. Materials and Methods

### 2.1. Study Areas

**Figure 1:**
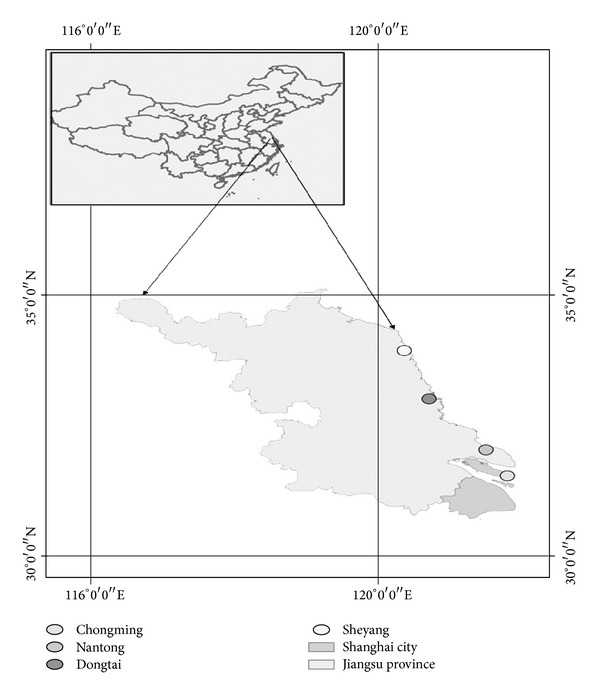
Locations of different sampling sites.

Four coastal wetlands were chosen in Sheyang, Dongtai, and Nantong of Jiangsu province and Chongming of Shanghai city (Figure 1). Each coastal wetland was divided into the bare beach,* Spartina* beach, and* Phragmites* beach according to vegetation type distribution.

Coastal zone of Jiangsu is affected by marine and continental climate. Average annual temperature is about 15°C. Average annual rainfall increases gradually from north to south, and average annual relative humidity decreases from south to north.

Chongming in Shanghai city is affected by subtropical marine monsoon climate. Average annual temperature is about 16°C, and average annual rainfall is about 1,030 mm.

### 2.2. Description of DNDC Model

The DNDC model takes denitrification and decomposition as the main processes applied in soil carbon and nitrogen biogeochemical cycles [14]. DNDC model has been applied in agriculture, forest, and grassland research worldwide for calculating soil carbon sequestration and greenhouse gas emissions [15]. This model consists of six submodels including soil, climate, plant growth, decomposition of organic matter, nitrification, denitrification, and fermentation process. Input variables of this model are soil properties, climate conditions, and agricultural production measures, and output variables are daily C and N content in soil and plant, soil temperature, and humidity data at different levels, and output variables are the emissions flux of CO_2_, CH_4_, N_2_O, and NO.

### 2.3. Acquisition of Meteorological Data

Daily observation meteorological data in 1988 (for 80s) and 2004 (for 00s) are obtained from “China Meteorological Data Sharing Service System” (http://cdc.cma.gov.cn/). According to input data requirements, the daily maximum temperature (°C), the daily minimum temperature (°C), the rainfall data (cm), and other correlated data are turned into text format (ASCII encoding) and ready for input into the model. Future meteorological data are calculated by IPCC simulations.

### 2.4. Collection of Soil Parameters

Land use type and soil texture in this study are wetlands and silt loam, respectively, which were set directly in the options of DNDC model. Soil bulk density, soil pH, and surface soil organic carbon (SOC) content are obtained from literatures and field measurements measured in 2004. By inputting these soil data, the model would give other corresponding soil data, and default data would be used in this study.

### 2.5. Collection of Plant Physiological Parameters

In this paper, plant types included no plant (bare beach),* Spartina,* and* Phragmites*, which were not listed in the DNDC model. Therefore, we chose “0 Fallow” of the DNDC model to simulate a bare beach, while* Spartina* and* Phragmites* were created as new plant options. The two plant physiological parameters including biomass, C∖N, LAI, and water requirement, for this model, were collected by literatures and field measurements in 2004.

### 2.6. Hydrological Data Preparation

The DNDC model provides four patterns to simulate the influence of flooding, including irrigation, rain-fed, observed water-table data, and empirical parameters. This study adopted observed water-table data pattern. Tidal data were obtained from tidal tables of the Yellow Sea and Bohai Sea, in 2004 and 1990, and Xiaoyang, Lvsi, Sheshan, and Sheyang port were chosen as tidal stations for Dongtai, Nantong, Chongming, and Sheyang, respectively. Every 10-day tidal height and tidal height datum of the port and the elevation of different wetlands were used to calculate the water levels of bare beach,* Spartina* beach, and* Phragmites* beach, which were made into water level table finally.

## 3. Results

### 3.1. Comparison of CH_4_ and N_2_O Annual Emission Flux of Different Beaches

**Figure 2:**
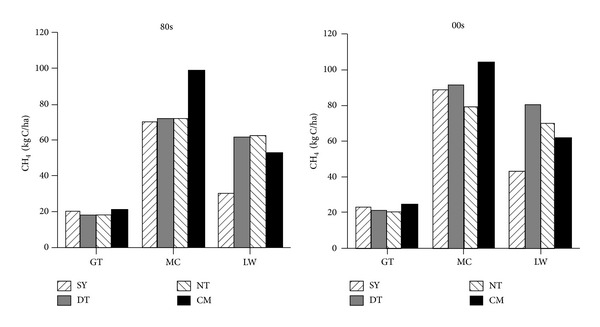
Comparison of CH_4_ emission flux of different beach in 80s and 00s (SY, DT, NT, and CM represent Sheyang, Dongtai, Nantong, and Chongming, resp.; GT, MC, and LW represent bare beach,* Spartina* beach, and* Phragmites* beach, resp.).

**Figure 3:**
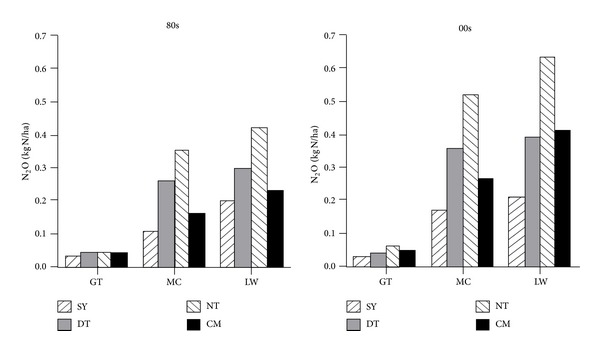
Comparison of N_2_O emission flux of different beach in 80s and 00s (SY, DT, NT, and CM represent Sheyang, Dongtai, Nantong, and Chongming, resp.; GT, MC, and LW represent bare beach,* Spartina* beach, and* Phragmites* beach, resp.).

The annual CH_4_ emission flux of different beaches including Sheyang, Dongtai, Nantong, and Chongming has a similar trend between 80s and 00s (Figure 2), and similar phenomenon occurred for N_2_O emission flux (Figure 3). The order of CH_4_ emission ability in different beaches is* Spartina *beach >* Phragmites* beach > bare flat, while for N_2_O the order is* Phragmites* beach >* Spartina* beach > bare flat (Figures 2 and 3).* Spartina alterniflora* and* Phragmites australis* increase CH_4_ and N_2_O emission fluxes contrasting with bare beach, which suggests that the vegetation type is one of the important factors affecting warm gas emission. CH_4_ emission ability of* Spartina *beach is higher than* Phragmites* beach, while N_2_O emission ability is contrary.

### 3.2. Comparison of Future CH_4_ and N_2_O Annual Emission Flux

**Figure 4:**
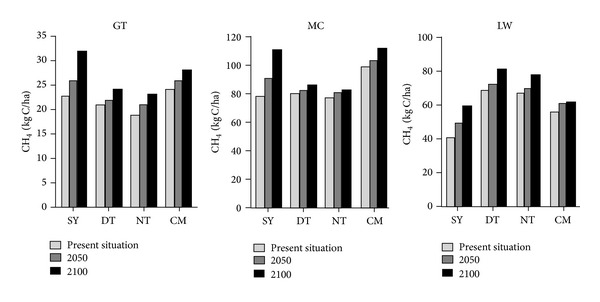
Comparison of present (average CH_4_ emission flux of 80s and 00s) and future CH_4_ emission flux of different vegetation zones at different locations.

**Figure 5:**
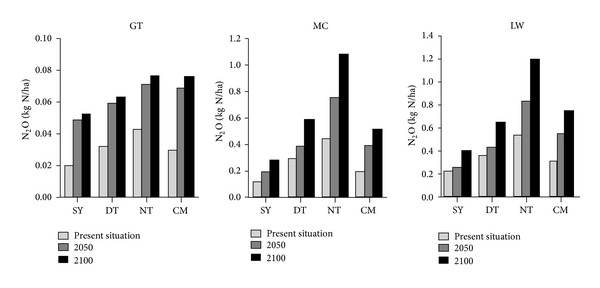
Comparison of present (average N_2_O emission flux of 80s and 00s) and future N_2_O emission flux of different vegetation zones at different locations.

**Figure 6:**
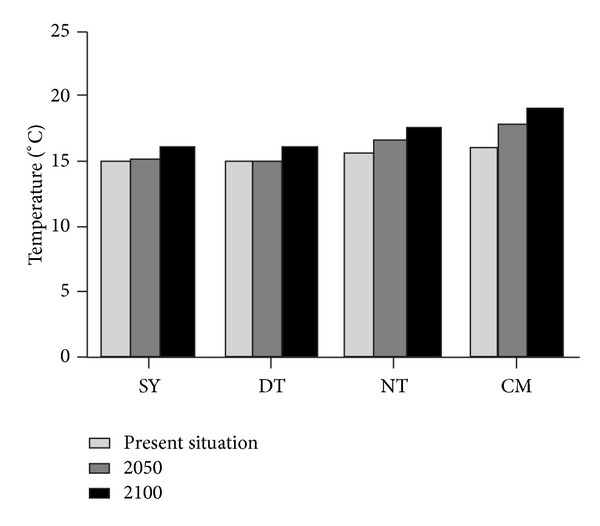
Comparison of present (average temperature of 80s and 00s) and future average annual temperature at different locations.

Based on Figures 4 and 5, CH_4_ and N_2_O emission flux of three different coastal wetlands increase with increasing time from the present situation to 2100 in four study sites, which is consistent with the predicted trend of rising temperature (Figure 6). For different beaches, CH_4_ emission flux of* Spartina* beach (MC) is >* Phragmites* beach (LW) > bare flat (GT), and N_2_O emission flux of* Phragmites* beach is >* Spartina* beach > bare beach.

In different sites, CH_4_ emission flux of CM and SY in MC and GT beach is higher than DT and NT, while on LW beach this trend is contrary. For N_2_O emission flux, NT is the highest while SY is the lowest.

### 3.3. Roles of Different Factors in CH_4_ and N_2_O Emissions

By correlation analysis, CH_4_ and N_2_O emission flux are significantly correlated with soil organic carbon content (*R* = 0.97,  *R* = 0.695  (*P* < 0.01), resp.) and with maximum biomass of per unit area (*R* = 0.822,  *P* < 0.01; *R* = 0.821, *P* < 0.01). Soil pH has a significant correlation with CH_4_ emission flux (*R* = 0.362, *P* < 0.05) and N_2_O emission flux (*R* = 0.402, *P* < 0.05). CH_4_ and N_2_O emission flux have significant negative correlation with average water level (*R* = −0.261, *P* < 0.05; *R* = −0.630, *P* < 0.01).

Study results show that CH_4_ emission flux in coastal wetland has significant correlation with temperature (*R* = 0.707, *P* < 0.01) and also is affected significantly by rainfall (*R* = 0.379, *P* < 0.05). N_2_O emission flux is positively correlated with temperature (*R* = 0.768, *P* < 0.01); however, N_2_O flux and total annual precipitation have no relevance.

## 4. Discussions

### 4.1. Roles of Similar Vegetation Distribution Zone in CH_4_ and N_2_O Emissions in Coastal Wetland along Latitude

Some studies showed that the emission of trace gases from wetlands was controlled by multiple factors like soil temperature, hydrology, and vegetation (e.g., [16–20]). Our study also indicated that vegetation, soil characteristics, and climate conditions affected emissions of CH_4_ and N_2_O in coastal wetlands. Moreover, it was noticeable that, at different sites along latitude, similar CH_4_ and N_2_O emission patterns were found, which was caused by similar vegetation distribution zone including bare beach,* Spartina* beach, and* Phragmites *beach in coastal wetlands. This indicated that vegetation was an important factor affecting greenhouse gas emission [21]. Van der Nat and Middelburg [22] found that gas emissions were affected collectively by vegetation type and species composition in tidal marshes, and increasing CH_4_ flux was achieved by improvement of organic substrates and transportation of plant aerenchyma reducing oxidation of methane [23, 24]. Windham and Ehrenfeld [25] pointed out that, in two different tidal areas dominated by reeds and cattails, CH_4_ emissions had some obvious differences. Exotic-species invasions affecting the vegetation structure of wetland ecosystems [25] would change the input of organic matter to soil [26]. Cheng et al. [9] reported that CH_4_ emissions were significantly correlated with plant biomass and stem density for* Spartina* and* Phragmites, *while N_2_O emissions were not accorded with this trend. Our model study also showed that, in a vegetation gradient zone, the effects of exotic-species* Spartina* on CH_4_ emissions were more than native species* Phragmites* for its higher biomass and organic matter. However, this phenomenon did not exist in N_2_O emission [27]. This was attributed to different mechanisms of CH_4_ and N_2_O emissions. Therefore, different vegetation distribution in coastal wetland was actually an important factor during the processes of CH_4_ and N_2_O emissions.

### 4.2. Effects of Temperature on CH_4_ and N_2_O Emissions

The changes between present and future CH_4_ and N_2_O emission flux in the same site were mainly due to future temperature higher than present temperature. Temperature, especially soil temperature, played a very important role in CH_4_ production and emission. Soil temperature directly affected the quantity, structure, and activity of a series of microflora that were involved in the process of the methane production and oxidation [7] and methane transport in soil [28]. Huttunen et al. [29] found that the correlation between CH_4_ emission flux and peat surface temperature was most significant in all factors in the northern peatland, and incubation experiments also proved this relationship [30]. Our study results also showed that CH_4_ emission flux in coastal wetland had significant correlation with temperature.

Hotness, humidity, and high carbon and nitrogen content of the soil were the best environment for N_2_O production. The temperature was an important environmental factor in soil N_2_O production and emission, but its relationship with N_2_O emission was still controversial. Luo et al. [31] argued that temperature affected soil nitrification and denitrification, and Dorland and Beauchamp [32] found that, while temperature varied from −2°C to 25°C, the amount of the square root of the denitrification had a linear relationship with temperature. However, some studies also suggested that the temperature impact was not obvious. This study was consistent with the result of [33] that N_2_O flux was positively correlated with temperature.

Comparisons of CH_4_ and N_2_O flux in different locations were complex. Multiple factors could cause the regional difference, such as temperature, rainfall, water level, and soil pH (e.g., [34, 35]).

## 5. Conclusions

By simulating CH_4_ and N_2_O emissions in the coastal wetlands by DNDC model, CH_4_ emission ability of* Spartina *beach in three different vegetation zones was the highest, while the highest N_2_O emission ability existed in* Phragmites* beach. This trend was very similar in the different sites including Sheyang, Dongtai, Nantong, and Chongming. By correlation analysis, CH_4_ and N_2_O emission fluxes were positively correlated with soil organic carbon content, temperature, and maximum biomass of per unit area and were negatively correlated with average water level. Comparisons of CH_4_ and N_2_O flux in different locations were complex, which were caused by multiple factors, such as temperature, rainfall, water level, and soil pH.
